# Adrenomedullin expression in epithelial ovarian cancers and promotes HO8910 cell migration associated with upregulating integrin α5β1 and phosphorylating FAK and paxillin

**DOI:** 10.1186/1756-9966-31-19

**Published:** 2012-03-09

**Authors:** Boya Deng, Siyang Zhang, Yuan Miao, Zhuang Han, Xiaoli Zhang, Fang Wen, Yi Zhang

**Affiliations:** 1 Department of Gynecology, The First Hospital of China Medical University, Shenyang 110001, China; 2 Center of Laboratory Technology and Experimental Medicine, China Medical University, Shenyang 110001, China; 3 Department of Pathology, The First Hospital and College of Basic Medical Sciences of China Medical University, Shenyang 110001, China; 4 Department of Orthopaedics, The First Hospital of China Medical University, Shenyang 110001, China

**Keywords:** Epithelial ovarian cancer, AM, Carcinogenesis, Progression, Migration, Integrin α5β1, FAK, Paxillin

## Abstract

**Background:**

Epithelial ovarian cancer (EOC) is one of the leading causes of cancer deaths in women worldwide. Adrenomedullin (AM) is a multifunctional peptide which presents in various kinds of tumors.

**Methods:**

In this study, we characterized the expression and function of AM in epithelial ovarian cancer using immunohistochemistry staining. Exogenous AM and small interfering RNA (siRNA) specific for AM receptor CRLR were treated to EOC cell line HO8910. Wound healing assay and flow cytometry were used to measure the migration ability and expression of integrin α5 of HO8910 cells after above treatments. Western blot was used to examine the phosphorylation of FAK and paxillin.

**Results:**

We found that patients with high AM expression showed a higher incidence of metastasis, larger residual size of tumors after cytoreduction and shorter disease-free and overall survival time. Exogenous AM induced ovarian cancer cell migration in time- and dose- dependent manners. AM upregulated the expression of integrin α5 and phosphorylation of FAK, paxillin as well.

**Conclusions:**

Our results suggested that AM contributed to the progression of EOC and had additional roles in EOC cell migration by activating the integrin α5β1 signaling pathway. Therefore, we presumed that AM could be a potential molecular therapeutic target for ovarian carcinoma.

## Background

Epithelial ovarian cancer (EOC) is the sixth most common cancer and the fifth leading cause of cancer mortality in women worldwide [1]. This lethal gynecological malignancy is commonly diagnosed at a late stage due to the silent early stage and easily metastasis. Many advances took place in the pathological study and in understanding the mechanisms involved in EOC progression, details still need further investigations [2,3]. Therefore, this is an urgent need of more effective and new molecular targeted therapies for EOC.

Adrenomedullin (AM) is a 52-amino-acid peptide first isolated from human pheochromocytoma [4]. It belongs to a family of peptides with calcitonin gene-related peptide (CGRP) and Amylin [5]. AM was identified as a major regulator of carcinogenesis and tumor progression, and autocrine loop of AM was targeted as new strategies against human cancers [6-8]. AM gene expression was proved to be associated with histological grade and poor prognosis of ovarian cancer [9]. The expression of its receptor calcitonin receptor-like receptor CRLR together with modulation factors RAMP2/RAMP3 were also found in EOC tissues and OVCAR3 cells [10,11]. Our previous study had found that AM was autocrined in EOC cell line CAOV3 by bFGF stimulation [12].Thus we supposed that AM may play an important role in EOC progression.

Integrins are family of transmembrane proteins, which are composed of 2 subunits as α- and β- formed heterodimer, and work as receptors of extracellular matrix (ECM) [13]. Integrins received and transmitted the signal from ECM into cells and modified various function of cells including shape, motility, and involved in EOC metastasis [14,15]. It was well accepted that integrin α5 specifically bound to integrin β1 to form specific receptor for fibronectin (FN). Activated integrin α5β1 could activate the focal adhension kinase (FAK) and Src, which consequently promoted cancer cells migration and invasion via activating various skeleton proteins, such as paxillin. It was reported that overexpression of integrin α5β1 predicted poor prognosis for EOCs [16]. And integrin α5β1 promoted ovarian cancer cells invasion by directly activating c-Met followed by FAK activation [17].

In this study, we evaluated AM expression in EOC tissues by immunohistological staining to analyze possible correlations between AM expression and the clinical determination of FIGO staging, differentiation, and prognosis. We further probed the biological features of AM by assaying cell migration and potential mechanism underlying this.

## Methods

### Cell culture and reagents

EOC cell line HO8910 cells were maintained in RPMI 1640 medium with 10% fetal bovine serum (Thermo Scientific Hyclone, USA), supplemented with 100 units/ml penicillin and 100 units/ml streptomycin (Sigma, USA) and maintained at 37°C with 5% CO_2_. When confluent, cells were treated with 100 nM AM (Phoenix Pharmaceuticals, USA). Cells were pretreated by 1 nM AM22-52 (Phoenix Pharmaceuticals, USA), or by 5 μg/ml the anti-integrin α5β1 monoclonal blocking antibody (mAb) (BD Biosciences, USA) 1 h followed by AM.

### Tissue samples

For immunohistochemical analysis, EOCs (n = 96) were collected from surgical specimens originating from the First Affiliated Hospital of China Medical University between 2000 and 2008. Clinical data were obtained from clinical databases and tumors were staged according to International Federation of Gynecology and Obstetrics (FIGO) guidelines. There were 82 cases that had complete follow-up records.

### Immunohistochemical staining and evaluation

All paraffin sections were deparaffinized and rehydrated. The sections were hematoxylin-and-eosin (HE) stained to confirm histological diagnosis by two pathologists (Yuan Miao and Xiaoli Zhang) according to the World Health Organization (WHO) classifications. Sections were subjected to antigen retrieval by heating in Tris-EDTA buffer at pH 8.0 in an autoclave sterilizer for 2 min. A blocking solution consisting of 3% H_2_O_2 _and 5% bovine serum albumin was used to block endogenous peroxidase activity and non-specific binding. The sections were incubated with goat anti-AM antibody (10 μg/ml; R&D, USA) overnight at 4°C. On the next day, the sections were treated with the secondary antibody and SP complex (streptavidin-peroxidase) for 40 min (Maixin Biotechnology, Fujian, China). Binding sites were visualized with 3,3'-diainobenzidine (DAB) after 1 min incubation (Maixin Biotechnology, Fujian, China). After counterstaining with Mayer's hematoxylin, the sections were dehydrated and mounted. For the negative control, phosphate-buffered saline (PBS) was used instead of the primary antibody.

We evaluated the cytoplasmic and membrane distribution of AM protein for statistical analysis in EOCs. One hundred cells were randomly selected and AM distribution was manually counted from 5 representative 400 × fields of each section by two independent observers (Yuan Miao and Boya Deng) in a blinded study. The percentage of cells positive for AM cytoplasmic and membrane expression was graded and counted as follows: (0 = negative; 1 = 1-50%; 2 = 50-74%; 3 ≥ 75%). The staining intensity score was graded as follows for cytoplasmic expression (1 = weak; 2 = intermediate; and 3 = strong). The scores for AM positivity and staining intensity were multiplied to obtain a final score, which determines AM expression as (- = 0; + = 1-2; ++ = 3-4; +++ = 6-9).

### Western blot

Cells were washed twice with ice-cold PBS, collected and homogenized on ice in 10 volumes (w/v) of lysis buffer containing 20 mM Tris-HCl, 1 mM EDTA, 50 mM NaF, 50 mM NaCl, 1 mM Na_3_VO_4_, 1% Triton X-100, AND 1 mM PMSF. The homogenate was centrifuged at 15,000 rpm for 30 min at 4°C. The supernatant was collected and protein content was determined using the BCA assay (Beyotime Institute of Biotechnology, Jiangsu, China). Protein was separated by 10% SDS-PAGE and then transferred to PVDF blotting membranes, which were then blocked for 2 h in 5% defatted milk in Tris-buffered saline containing Tween-20 (TBST, 10 mM Tris-HCl, 150 mM NaCl, 0.1% Tween-20). For immunoblotting, the membrane was incubated at 4°C overnight with anti-β-actin (1:1000, Keygen Biotech, China), anti-CRLR (1:1000, Phoenix, USA), anti-FAK (1:500), anti-FAK pY397 (1:500), anti-paxillin (1:500), anti-paxillin pY118 (1:500), which were all from Santa Cruz company (Santa Cruz, USA). Then, it was rinsed with TBST three times and incubated with corresponding horseradish peroxidase conjugated IgG antibodies (1:2000, Zhongshan Golden Bridge Biotechnology, Beijing, China) for 2 h. Immunoreactive bands were visualized using ECL (Beyotime Institute of Biotechnology, Jiangsu, China). The MF-ChemiBIS 3.2 Imaging System (DNR Bio-Imaging Systems, Israel) was used for image capture. The optical density (OD) of each band was measured using Image J software.

### Migration assay

Cells were plated on 24 well-plates at 5 × 10^4^/well. The next day, cells were washed with PBS and wounds were created by scraping with a sterilized pipette tip. After washed twice with PBS, cells were incubated in RPMI-1640 containing 0.5% fetal bovine serum. The wound closure was monitored at 0-12 h. The wound areas were observed by an inverted microscope (OlympusIX71, Japan) and measured by Image J at the exact place and the healing percentages were calculated. Each test was performed triplicates.

### CRLR knockdown with siRNA

The CRLR-specific small interfering RNA (siRNA) (#42272) and scrambled siRNA (#4611) were designed and synthesized by Ambion (USA). Using Lipofectamine 2000 (Invitrogen, CA, USA), HO8910 cells were transfected with siRNAs following the manufacturer's protocol. Cells were cultured with fresh medium 6 h after transfection.

### Real-time PCR

To confirm the effection of siRNA, we carried out real-time RT-PCR by using SYBR Premix Ex Taq™ II kit (Takara, Japan). Total RNA was extracted by RNAiso Plus (Takara, Japan) according to the manufactor's protocol. 2 microgram of total RNA were subjected to cDNA synthesis by AMV transctriptase and the random primer (Takara, Otsu, Japan). Oligonucleotide primers for *CRLR *were designed as follows: forward: 5'-GGATGGCTCTGCTGGAACGATGT -3' and reverse: 5'-TGCAGTCTTCACTTTCTCGTGGG -3' (204 bp). The primers for the internal control, β-actin were forward: 5'- AAGGCTGTGGGCAAGG -3' and reverse: 5'-TGGAGGAGTGGGTGTCG -3' (238 bp). PCR amplification of cDNA was performed in 20 μL mixtures containing 10 μL SYBR Premix Ex Taq (×2) with 0.08 μL of each primer, 0.4 μL of ROX Reference Dye, and 1 μL of template cDNA (50 μg/μL). The protocol included the following parameters: an initial 30 s of incubation at 94°C followed by 40 cycles of denaturation at 95°C for 5 s and annealing at 60°C for 35 s. Each experiment was done at least in triplicate, and the gene expression levels were calculated by *ΔΔ*Ct method.

### Flow cytometer analysis

To study the cell surface expression of integrin α5 anti-integrin α5 mAb (IIA1) (BD Biosciences, USA) were used at the recommended concentrations [18]. Cells were incubated with antibody for 30 min at 4°C and washed with PBS 3 times. Then cells were incubated with PE-conjugated IgG (1:300, Beijing Zhongshan Golden Bridge Biotechnology Co. China) for 45 min at 4°C, washed and fixed in 2% formaldehyde. Cells immunofluorescent contents were evaluated with a FACSCalibur flow cytometer (BD Biosciences, USA).

### Statistical analysis

SPSS 16.0 software was employed for all data analysis. Statistical evaluation was performed using the Spearman correlation test to analyze the rank data between the AM expression and clinicopathological parameters. Overall and disease-free survival curves were generated using the Kaplan-Meier method, and the differences between the curves were assessed using the Log-rank test. A COX proportional hazard model was used to determine the factors related to survival time. And one-way ANOVA was used to analyze the wound healing rates between groups and realtime PCR results as well. *P *< 0.05 was considered as statistically significant.

## Results

### Clinical significance of AM expression in ovarian carcinomas

**Figure 1:**
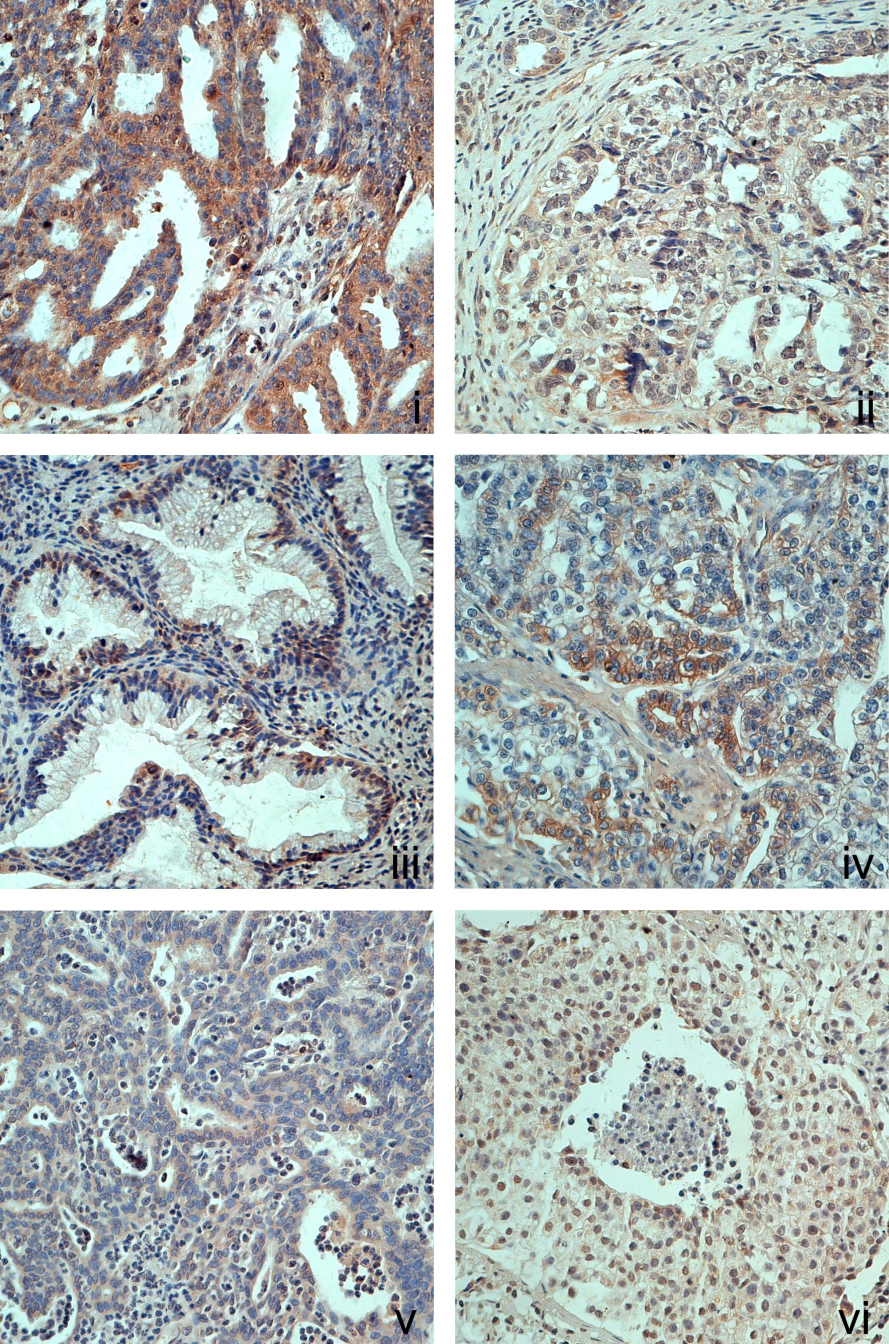
**AM expression in EOC samples**. Immunohistochemical analysis of AM expression in EOCs. EOCs: FIGO III stage serous (i), FIGO I stage serous (ii), mucinous (iii), clear-cell (iv), endometrioid (v) ovarian cancer, malignant Brenner tumor of ovary (vi).

There were 96 EOC cases eligible for our study. The age of patients ranged from 30 to 77 years (median = 52). Of all the cases, 17 were FIGO-I ovarian carcinomas, 19 were FIGO-II stage, 53 were FIGO-III stage and 7 were FIGO-IV stage. AM was mainly expressed in the cytoplasm and membrane of EOCs, seldom in nuclear of EOC cells, and was also expressed in the endothelial vessel cells and stromal cells in tumors, as shown in Figure 1 using immunostaining. In ovarian malignant tumor samples, 91.67% of cases (88/96) showed AM protein expression in the membrane and the cytoplasm of EOCs. As shown in Table 1, AM expression was positively correlated with FIGO stage (*P = *0.003), residual tumor after initial laparotomy (*P *= 0.000), but not with age, degree of differentiation, or serum CA125 before operation.

**Table 1:** Relationship between AM expression and clinicopathological features in EOCs

Clinicopathological features	n	AM expression				
		
		-	+	++	+++	*P *value
Age(years)						0.705
< 55	56	5	18	12	21	
≥ 55	40	3	9	9	19	
Histotype						0.155
Serous	75	7	17	17	34	
Non serous	21	1	10	4	6	
Residual tumor after initial laparotomy						0.000*
< 1 cm	42	1	21	10	10	
≥ 1 cm	53	7	6	11	29	
Undetermined	1	0	0	0	1	
Differentiation						0.199
Well-differentiated	27	4	11	6	6	
Moderately-differentiated	23	1	8	3	11	
Poorly-differentiated	39	3	7	11	18	
Undetermined	7	0	1	1	5	
FIGO staging						0.003*
I	17	1	10	1	5	
II	19	0	10	4	5	
III	53	6	7	13	27	
IV	7	1	0	3	3	
Serum CA125						0.301
≥ 500	52	5	11	13	23	
< 500	41	3	16	7	15	
Undetermined	3	0	0	1	2	

*P < 0.05

EOC = epithelial ovarian cancer; AM = Adrenomedullin; FIGO = International Federation of Gynecology and Obstetrics

**Figure 2:**
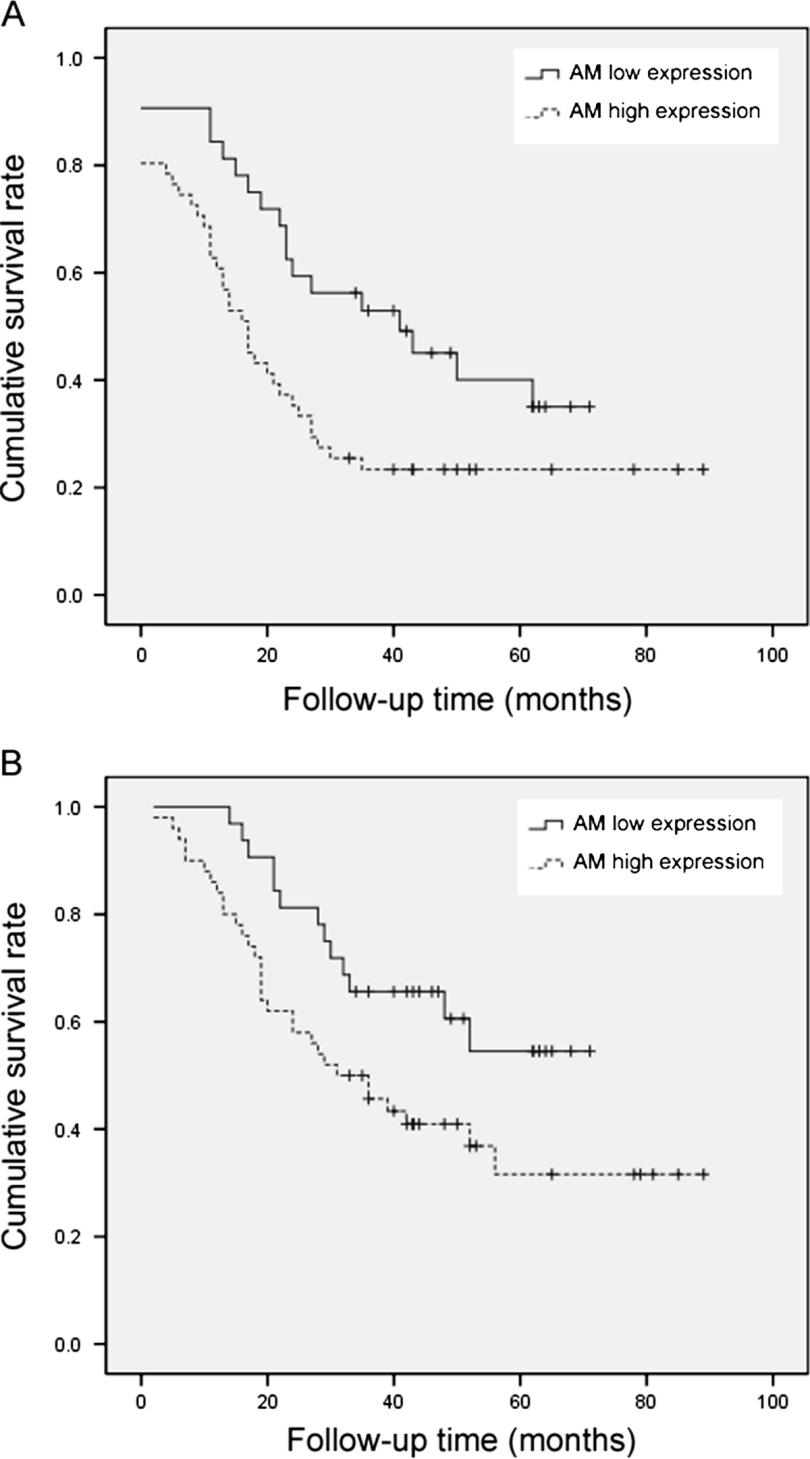
**Correlation between AM status and EOC patient prognosis**. Kaplan-Meier curves for disease-free survival rate of EOC patients according to the AM expression status (A); Kaplan-Meier total survival curves of EOC patients according to the AM expression status (B). Both survival time and disease-free time of patients were linked to AM expression status (Log-rank test, *P *= 0.020; *P *= 0.030).

Follow-up information was available for 82 EOC patients with survival periods ranging from 2 to 89 months (median = 36 months). Survival curves for EOCs were stratified according to AM expression (Figure 2). By using the *Kaplan-Meier *method, we indicated that both survival time and disease-free time for patients were linked to AM expression status (disease-free time, *P *= 0.020, Figure 2A; overall survival time, *P *= 0.030, Figure 2B). By using univariate Cox proportional analysis, AM expression was statistically correlated to disease-free survival and overall survival (*P *< 0.05, Table 2). By using multivariate Cox proportional analysis, considering all the clinical parameters and AM expression, FIGO staging was an independent factor of disease-free survival prognosis prediction, and both age and disease-free time were independent factors predicted EOC over-all survival prognosis (*P *< 0.05, Table 3).

**Table 2:** Univariate Cox proportional hazards regression analyses of clinicopathological variables and AM expression for EOC patient outcome

	Disease-free survival		Overall survival	
Variables				
	
	Relative risk (95%CI)	*P *value	Relative risk (95%CI)	*P *value
Age(≥ 55 years)	1.663(0.985-2.808)	0.057	2.174(1.201-3.935)	0.010*
Differentiation	1.542(1.084-2.193)	0.016*	1.449(0.971-2.161)	0.069
FIGO staging(II-IV)	4.883(1.937-12.309)	0.001*	5.285(1.630-17.131)	0.006*
Residual tumor after				
initial laparotomy (≥ 1 cm)	2.776(1.598-4.824)	0.000*	2.760(1.458-5.227)	0.002*
AM expression	1.878(1.081-3.265)	0.025*	2.014(1.052-3.852)	0.035*
Disease-free time			0.925(0.904-0.946)	0.000*

*P < 0.05, *P *value were calculated by Wald statistics

**Table 3:** Multivariate Cox proportional hazards regression analyses of clinicopathological variables and AM expression for EOC patient outcome

	Disease-free survival		Overall survival	
Variables				
	
	Relative risk (95%CI)	*P *value	Relative risk (95%CI)	*P *value
Age(≥ 55 years)	1.663(0.983-2.813)	0.058	1.880(1.012-3.495)	0.046*
Differentiation	1.061(0.785-1.434)	0.702	0.964(0.689-1.349)	0.830
FIGO staging(II-IV)	4.886(1.938-12.322)	0.001*	0.949(0.219-4.118)	0.944
Residual tumor after				
initial laparotomy (≥ 1 cm)	1.514(0.794-2.888)	0.208	1.285(0.651-2.537)	0.469
AM expression	1.307(0.735-2.324)	0.362	0.868(0.426-1.769)	0.697
Disease-free time			0.927(0.906-0.948)	0.000*

*P < 0.05, *P *value were calculated by Wald statistics.

CI = confidence interval.

### AM promoted ovarian cancer cells migration

**Figure 3:**
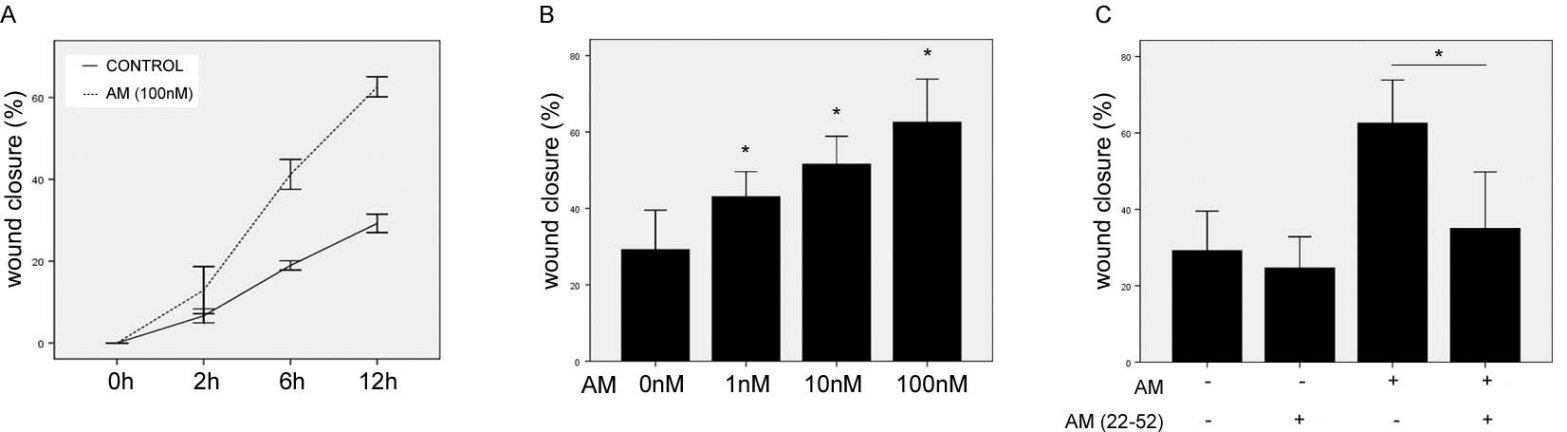
**Enhanced migration by AM in time-dependent and dose-dependent manners**.

**Figure 4:**
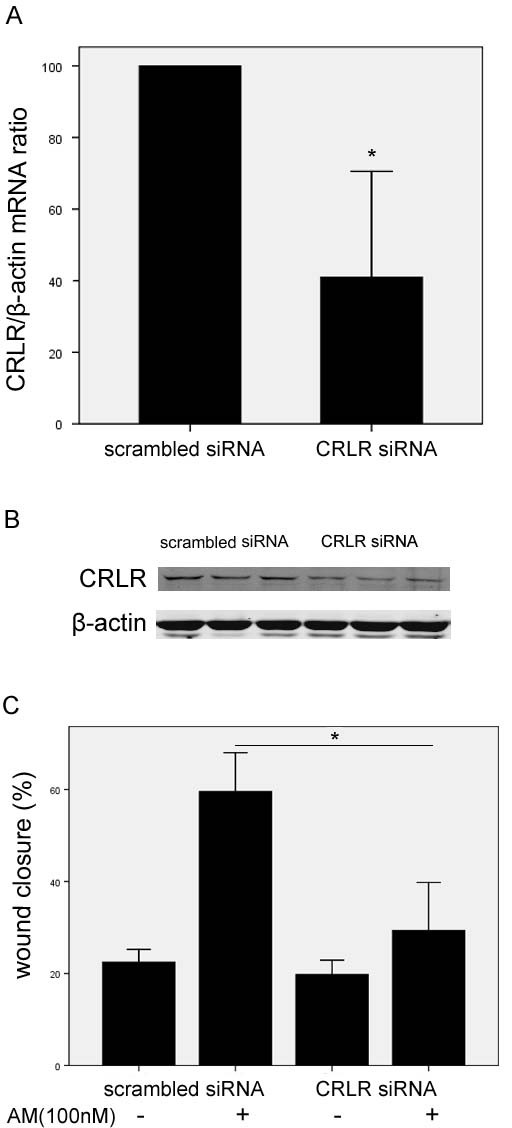
**Down-regulation of CRLR expression in HO8910 cells inhibited influence of exogenous AM on cell migration**. Reduced CRLR mRNA expression (A) and protein expression (B) were determined by real-time PCR analysis or western blot in CRLR siRNA transfected cells, compared with scrambled siRNA transfected cells. After cells were transfected by CRLR siRNA, the effect of AM on cells migration was decreased consequently (C).

HO8910 cells migration was enhanced with exogenous AM treatment in both dose-dependent and time dependent manners, as shown in Figure 3. Cell migration rates were consequently increased when cells were treated with different dose of AM (1, 10, 100 nM) for 12 h (Figure 3A). Recovery rates were 29.23 ± 4.15% with negative control, 43.06 ± 2.63% with 1 nM (*P *= 0.008), 51.58 ± 2.93% with 10 nM (*P *= 0.002),62.61 ± 4.51% with 100 nM (*P *= 0.001), respectively. A time course experiment was provided with AM (100 nM) by different incubation periods (1 h, 6 h, and 12 h). And the AM effect was increased gradually at 2 h (*P *= 0.023), and reached the maximum at 12 h (*P *= 0.000, Figure 3B). AM22-52, the receptor antagonist of AM, inhibited HO8910 cell migration (*P *= 0.024), and significantly inhibited the effect of AM on the migration of cells (*P *= 0.015, Figure 3C). Previously knockdown of AM receptor CRLR by siRNA effectively aborted the expression of mRNA (*P *= 0.013, Figure 4A) and protein expression of CRLR in HO8910 cells (Figure 4B). When cells were transfected with CRLR siRNA, the effect of AM on cell migration was decreased consequently (*P *= 0.001, Figure 4C).

HO8910 cells were treated with exogenous AM (100 nM) before subjecting to cell migration assay. Wound healing percentages were measured and calculated at time point of 3 h, 6 h, 12 h (A). Different concentration of AM (1, 10, 100 nM) were administrated to HO8910 cells and wound healing percentages were calculated at 24 h (B). AM (22-52) inhibited HO8910 cells migration and also antagonized the AM (100 nM) effect on migration (C). Each test was repeated triplicates

### AM enhanced HO8910 cell migration was linked to the activation of integrin α5β1 signaling pathway

**Figure 5:**
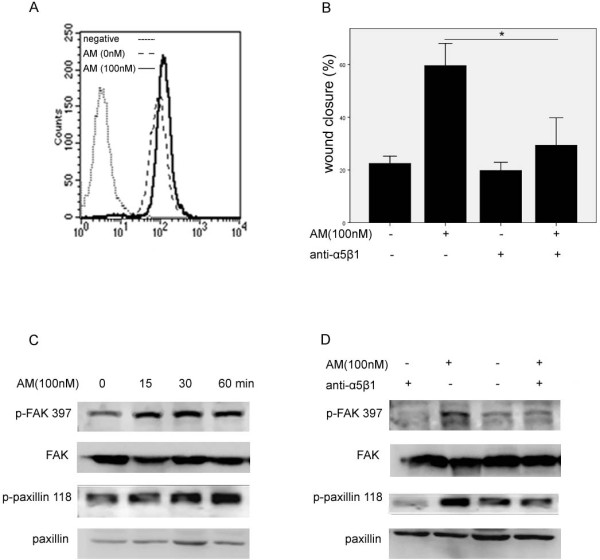
**Exogenous AM promoted cell migration with increased integrin α5β1 activation**. FACS flow analysis showed increased expression of integrin α5 in AM treated HO8910 cells than in non-treated cells (A). Blocking antibody of integrin α5β1 inhibited the effect of AM on cell migration (B). Exogenous AM promoted FAK and paxillin phosphorylation at different time point (C). Blocking antibody of integrin α5β1 abolished the AM promotion on FAK, paxillin phosphorylation (D).

By using flow cytometry, we studied the effects of AM on the expression of integrin α5. At 12 h after providing AM (100 nM), significant increased integrin α5 expression was observed in AM treated cells (Figure 5A). We found that the blocking antibody against integrin α5β1 effectively downregulated the cell migration promotion effects of AM (P = 0.000, Figure 5B). We also found that AM induced the phosphorylation of FAK and paxillin. Treatment with AM (100 nM) significantly increased the phosphorylation status of FAK 397 at 15 min time point, and paxillin 118 at 60 min (Figure 5C). And blocking the integrin α5β1 activity significantly inhibited the phosphorylation of FAK and paxillin by AM (Figure 5D).

## Discussion

AM is a peptide and pathologically elevated in various tumors. We described the relationship between AM expression and clinicopathological parameters of 96 cases of EOC with immunohistochemical analysis in the present study. We found that AM expression was positively related to the FIGO stage and with residual tumor size after initial surgical treatment. These data indicated that expression of AM might contribute to more aggressive behavior of EOC, and participate in EOC progression. AM high expression showed shorter disease free time and over-all survival time, which was similar with Hata's research by analyzing AM mRNA expression in 60 cases of EOCs [9]. We separately evaluated prognostic value of various factors by univariate COX proportional analysis, and found that AM expression was significantly associated with both the disease free survival and over-all survival. By using multivariant COX proportional analysis which evaluated all variants together, FIGO staging and age were independent factors of EOC prognosis prediction.

In order to further investigate the effects of AM on EOC progression, we provided exogenous AM to EOC cell line HO8910. The migratory rate of HO8910 was significantly increased in AM treated groups, which was blocked by the receptor antagonist AM22-52. Then, we endogenously decreased the AM receptor CRLR expression by specific siRNA, and found that CRLR downregulation mostly blocked the positive effect of AM on cell migration. Thus we considered that CRLR played crucial roles in AM promoting migration of HO8910 cells.

In this study, we also observed that AM significantly increased integrin α5 expression by FACS analysis, indicating a new signaling for AM function. Antibodies of integrin α5β1 were mainly used to anti-tumors treatment [19,20], especially for the advanced platinum-resistance EOCs [21]. In this study, the blocking antibody was used to illustrate whether integrin α5β1 was involved in AM induced cell migration. We found that cell migratory rate was decreased in integrin α5β1 antibody pretreated group compared to single AM treated cells. Also, factors associated with integrin α5β1 were analyzed in our study. Integrin α5β1 could consequently activate many cytoskeleton proteins by binding to FN, of which FAK and paxillin were crucial members [22-24]. It was shown that FAK phosphorylation was required for integrin stimulated cell migration by creating a binding site for the Src kinase family. FAK could also phosphorylate paxillin by in vitro and in vivo studies [25,26]. Paxillin was a cytoskeletal component involved in integrin signals integration and dissemination. Phosphorylation of paxillin greatly enhanced its function during cell migration [27,28]. Our study showed that exogenous AM treatment enhanced phosphorylation of FAK Tyr397 and paxillin Tyr118. The blocking antibody for integrin α5β1 mostly inhibited the AM induced upregulation of FAK and paxillin phosphorylation as well. Therefore, in our research, AM promoted HO8910 cells migration probably by upregulating expression of integrin α5β1 and increasing FAK and paxillin phosphorylation. However, the mechanisms of AM affection on integrin α5β1 needs further investigation, which might be owing to the enhanced integrin-binding function of talin by AM [29].

## Conclusions

In the summary, we found that high expression of AM contributed to the progression of EOC and indicated poorer prognosis of EOC patients, which further demonstrated its contribution to EOC metastasis probably via integrin α5β1 mediated cell migration. All of which suggested that AM might play great roles during EOC cell migration, and might be considered as an EOC therapeutic target.

## Competing interests

The authors declare that they have no competing interests.

## Authors' contributions

By D initiated the research, carried out the experiments and wrote the manuscript, YZ helped with the experimental design and gave funding support, SyZ, YM, ZH and XlZ gave experimental instructions, and FW gave critical review of the manuscript. All authors read and approved the final manuscript.
